# Artificial Intelligence and Neurosurgery: Tracking Antiplatelet Response Patterns for Endovascular Intervention

**DOI:** 10.3390/medicina59101714

**Published:** 2023-09-25

**Authors:** Khushi Saigal, Anmol Bharat Patel, Brandon Lucke-Wold

**Affiliations:** 1 College of Medicine, University of Florida, Gainesville, FL 32610, USA; 2 College of Medicine, University of Miami—Miller School of Medicine, Miami, FL 33136, USA; anmol.patel@med.miami.edu; 3 Department of Neurosurgery, University of Florida, Gainesville, FL 32608, USA

**Keywords:** artificial intelligence, antiplatelet therapy, endovascular intervention

## Abstract

Platelets play a critical role in blood clotting and the development of arterial blockages. Antiplatelet therapy is vital for preventing recurring events in conditions like coronary artery disease and strokes. However, there is a lack of comprehensive guidelines for using antiplatelet agents in elective neurosurgery. Continuing therapy during surgery poses a bleeding risk, while discontinuing it before surgery increases the risk of thrombosis. Discontinuation is recommended in neurosurgical settings but carries an elevated risk of ischemic events. Conversely, maintaining antithrombotic therapy may increase bleeding and the need for transfusions, leading to a poor prognosis. Artificial intelligence (AI) holds promise in making difficult decisions regarding antiplatelet therapy. This paper discusses current clinical guidelines and supported regimens for antiplatelet therapy in neurosurgery. It also explores methodologies like P2Y12 reaction units (PRU) monitoring and thromboelastography (TEG) mapping for monitoring the use of antiplatelet regimens as well as their limitations. The paper explores the potential of AI to overcome such limitations associated with PRU monitoring and TEG mapping. It highlights various studies in the field of cardiovascular and neuroendovascular surgery which use AI prediction models to forecast adverse outcomes such as ischemia and bleeding, offering assistance in decision-making for antiplatelet therapy. In addition, the use of AI to improve patient adherence to antiplatelet regimens is also considered. Overall, this research aims to provide insights into the use of antiplatelet therapy and the role of AI in optimizing treatment plans in neurosurgical settings.

## 1. Introduction

Platelets play a crucial role in the maintenance of normal blood clotting and the formation of blood clots. The process of platelet aggregation is a significant factor in the development of various arterial blockages such as coronary artery disease, strokes, and peripheral arterial disease. Consequently, the use of medications that inhibit platelet function, known as antiplatelet therapy, is essential in preventing recurring events in individuals affected by these conditions [1,2,3,4].

There is a lack of comprehensive guidelines regarding the use of antiplatelet agents in elective neurosurgery. The decision to continue antiplatelet therapy during surgery presents a risk due to increased bleeding, while discontinuing the medication prior to surgery carries the risk of thrombosis. In general, the discontinuation of antiplatelet therapy is typically the recommended course of action in neurosurgical settings [5], but this carries an elevated risk of ischemic events, including potentially life-threatening stent thrombosis, myocardial infarction, and stroke. Conversely, maintaining antithrombotic therapy may heighten the chances of bleeding and the need for blood transfusions, both of which are recognized factors that contribute to a poor prognosis [6]. It is possible that these difficult decisions regarding when to use antiplatelet therapy could be determined by artificial intelligence (AI) through utilizing technology to monitor individual antiplatelet compliance and platelet function.

Artificial intelligence (AI) is increasingly shaping medical decision-making, with notable applications in neurosurgery and antiplatelet therapy. In neurosurgery, AI aids in assessing the delicate balance between ischemia and bleeding risks post-stent placement or -WEB embolization. Although most AI research has historically focused on cardiovascular medicine, recent studies have developed AI models for predicting ischemic and bleeding risks in patients undergoing drug-eluting stent implantation. Additionally, AI can enhance medication adherence through smartphone applications, ensuring that patients follow their prescribed regimens, including antiplatelet therapies. The integration of AI in medical decision-making holds promise for optimizing individualized treatment plans in the complex realm of neuroendovascular procedures.

Hence, in this paper, the current clinical guidelines on the indications for antiplatelet therapy in neurosurgery will be discussed as well as supported antiplatelet regimens for such indications. In addition, current methodologies which can be used to decide when to use such antiplatelet regimens, such as P2Y12 reaction units (PRU) monitoring and thromboelastography (TEG) mapping, will be discussed. Finally, this paper discusses the use of AI, which can solve several of the limitations posed by PRU monitoring and TEG mapping, and its aid in making decisions regarding when to use antiplatelet regimens will be considered.

## 2. Materials and Methods

This paper is a comprehensive literature review searching PubMed and Google Scholar with no specific time period limitations. For information on the indications of antiplatelet regimens in neurosurgery, the keywords “antiplatelet”, “indication”, and “neurosurgery/”neuroendovascular” were used. For information on how antiplatelet therapy is currently monitored, the keywords “antiplatelet”, “therapy”, “PRU monitoring”/”TEG mapping”, and “neurosurgery”/“neuroendovascular” were used. To study how artificial intelligence has been used to monitor antiplatelet therapy, the keywords “antiplatelet”, “therapy”, “artificial intelligence”/“machine learning”, and “neurosurgery”/“neuroendovascular” were used. To study how artificial intelligence has been used to predict hemorrhage/ischemia, the keywords “artificial intelligence”/ “machine learning”, “ischemia”, “hemorrhage”, and “neurosurgery”/”neuroendovascular” were used.

## 3. Results

### 3.1. Indications for Antiplatelet Therapy

#### 3.1.1. Stents

In neurosurgical interventions, antiplatelet therapy is used in the setting of endovascular stent placement for either unruptured or ruptured aneurysms and/or arteriovenous malformations. Bare metal stents typically become fully endothelialized within a period of four–six weeks, which is when the risk of thrombosis is the highest. The abrupt discontinuation of antiplatelet therapy further increases this risk [7]. To minimize the occurrence of blood clot-related complications linked to the insertion of neurovascular stents, the utilization of DAPT has become a customary approach in recent years [8,9,10,11], with the majority of patients initiating DAPT at least 5 days, usually 14, prior to the procedure [11]. The management of pharmacologic therapy using antiplatelet agents during the endovascular treatment of a ruptured aneurysm varies, and there is a lack of high-quality data to guide management decisions. Some authors suggest that managing ruptured aneurysms can be similar to managing unruptured aneurysms, with the addition of a ventriculostomy if needed to manage a subarachnoid hemorrhage. The preferred approach during the procedure involves a dual antiplatelet regimen comprising aspirin and an intravenous glycoprotein IIb/IIIa inhibitor, followed by a switch to oral medications afterward [12]. A systematic review of stent-assisted coiling for ruptured brain aneurysms revealed that delaying the administration of antiplatelet agents after the procedure increased the risk of thromboembolic events, which differed from the practice of administering antiplatelet agents before and during the procedure. The latter approach resulted in a thromboembolic risk similar to that of the stent coiling of unruptured aneurysms [13]. Another retrospective study on the stent-assisted coiling of ruptured brain aneurysms described the use of dual antiplatelet therapy (DAPT) (aspirin and clopidogrel) loaded immediately before the procedure, intravenous heparin during the procedure, intra-arterial tirofiban if stent thrombosis was observed, and single or DAPT following the procedure. The reported event rates were comparable to a group of patients undergoing primary coiling alone, ranging from 1.4% for rebleeding and post-procedural cerebral infarction to 2.8% for intra-procedural thrombotic events [14]. Several reports in the literature suggest a decrease in thromboembolic events with the use of clopidogrel. However, the significance of adding aspirin in the post-procedure setting is not well understood, and many practitioners choose to use aspirin instead of clopidogrel [10,15,16,17]. For the treatment of unruptured aneurysms, it is important to consider various scenarios such as primary coil embolization, coiling with balloon assistance, stent-assisted coiling, intrasaccular flow disruptor placement, and parent artery flow diversion using braided stents. Based on increasing experience with flow diverters over the past decade, many practitioners have adopted DAPT guided by point-of-care testing for most of the aforementioned scenarios, except for primary coil embolization. A recent meta-analysis of 1005 patients undergoing intracranial flow diversion concluded that DAPT including ticagrelor or prasugrel is safe, and ticagrelor use may be associated with better survival than clopidogrel use [18].

Some researchers have treated arteriovenous malformation (AVM)-associated feeding artery aneurysms with flow-diverting stents [19,20,21] or stent-assisted coiling [22] in small case studies. These studies have advocated for the use of a standard DAPT to prevent stent thrombosis. However, there was insufficient follow-up duration to assess whether this approach increases the risk of intracranial hemorrhage compared to the baseline risk for AVMs when receiving radiosurgery or non-interventional surveillance [23]. While there are new trials that potentially allow for variations in the current medical practice of antiplatelet regimens in intracranial atherosclerotic disease (ICAD), the majority of patients undergoing balloon angioplasty and/or stenting still receive DAPT before and after the procedure. Non-randomized studies, such as the WEAVE trial (Wingspan Stent System Post-Market Surveillance), provide guidance on post-intervention antiplatelet therapy. In this trial, the rate of complications during the procedure was relatively low at 2.6%. The DAPT regimen consisted of aspirin plus a P2Y12 inhibitor for 7–10 days before stenting and for 90 days after stenting [24]. After 90 days, the regimen was switched to aspirin alone. This protocol is similar to those used in other intracranial stenting procedures and is considered an acceptable practice in the management of ICAD [23].

#### 3.1.2. WEB Embolization

**Figure 1:**
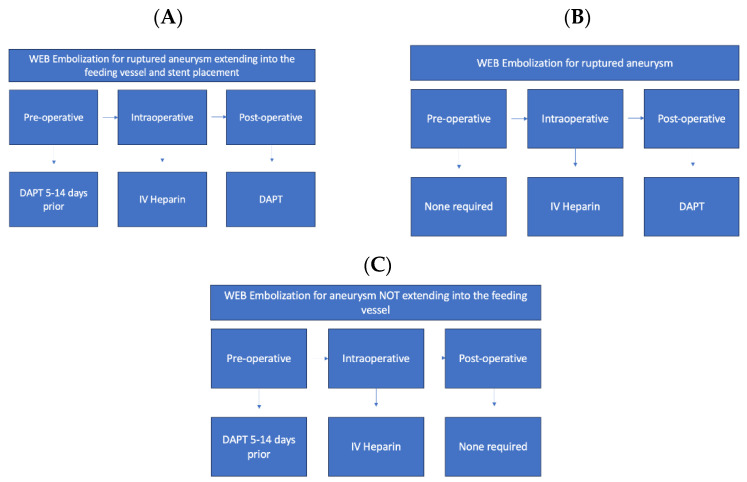
Antiplatelet and anticoagulant regimens for stent and WEB Embolization (**A**). Antiplatelet and anticoagulant regimen in the pre-, intra-, and post-operative stages when utilizing stent or web-embolization for unruptured aneurysm that extends into the feeding vessel; (**B**) antiplatelet and anticoagulant regimen in the pre-, intra-, and post-operative stages for WEB embolization for ruptured aneurysm; (**C**) antiplatelet and anticoagulant regimen in the pre-, intra-, and post-operative stages for WEB embolization for aneurysm that does not extend into the feeding vessel.

In addition, antiplatelet therapy is often necessitated post-neurosurgical intervention in the setting of the Woven EndoBridge (WEB) Aneurysm System. The WEB Aneurysm System is a nitinol self-expanding mesh used to treat saccular, wide neck, or bifurcation intracranial aneurysms to promote thrombosis with occlusion. The pre-treatment and post-treatment use of antiplatelets is debated and typically dependent on clinical pictures. In ruptured aneurysms, pre-treatment is typically not administered. If the entire WEB system is contained within the aneurysm, post-treatment antiplatelet therapy may not be indicated unless the system extends into the artery or if the aneurysm has a large neck due to the risk of thrombosis formation into a feeding artery [25,26]. For unruptured aneurysms, pre-treatment with DAPT is considered to allow for the use of stents if necessary, without increased thrombosis risk. Post-treatment DAPT is not indicated [27]. Figure 1 demonstrates antiplatelet and anticoagulant regimens for stent and WEB embolization for unruptured aneurysms (Figure 1A), ruptured aneurysms (Figure 1B), and for aneurysms which do not extend into the feeding vessel (Figure 1C).

## 4. Antiplatelet Regimens

### 4.1. Aspirin

Aspirin is a frequently used component of DAPT for patients undergoing neuroendovascular procedures. Aspirin has an onset of 7–60 min and a duration of action of three–five days. Typically, aspirin is initiated 3–5 days prior to carotid or intracranial interventions, although some practitioners recommend starting therapy as early as 14–21 days before the procedure. In emergency neuroendovascular interventions where preprocedural aspirin therapy is not possible, loading doses have varied from 200 to 650 mg. Almost all studies examining antiplatelet therapy in neuroendovascular procedures include aspirin as part of the regimen, with daily doses ranging from 81 to 325 mg [11].

### 4.2. Clopidogrel

Clopidogrel is another commonly used component of DAPT for patients undergoing neuroendovascular procedures. It has an onset of 2 h and a duration of action of approximately three–seven days. Clopidogrel, as a second-generation blocker of the adenosine diphosphate (ADP) P2Y12 receptor, needs to undergo conversion into an active form to exert its antiplatelet effects. This activation process may cause a delay in its antiplatelet properties, which is why a loading dose is often administered to achieve the maximum inhibition of platelet function [28,29].

### 4.3. Aspirin and Clopidogrel

Combining aspirin and clopidogrel results in a greater inhibition of platelet function compared to using either medication alone. The duration of therapy for neuroendovascular procedures tends to be longer than that for bare metal stent deployment in percutaneous coronary intervention (PCI), with most practitioners prescribing DAPT for at least three months [11]. Clinical trials and meta-analyses have shown that the combination of aspirin and clopidogrel slightly reduces the recurrence of ischemic stroke but significantly increases the risk of bleeding events when compared to using aspirin or clopidogrel individually [30,31,32,33]. Therefore, applicable guidelines advise against the use of DAPT (such as aspirin and clopidogrel) for preventing secondary stroke in patients in the chronic phase of noncardioembolic ischemic stroke [34].

### 4.4. Clopidogrel Non-Responders

In a small subset of the population, clopidogrel or DAPT with aspirin and clopidogrel do not function as expected. Clinically, this is defined as having an atherothrombotic event while on antiplatelet therapy or as determined by a lab test such as PRU or TEG [35]. One study found that 15.5% of those that are compliant with DAPT were non-responders to DAPT with aspirin and clopidogrel [36]. For some, this is due to factors such as non-compliance or interaction with other medications [37]. For others, it can be due to increased platelet activity, problems with absorption, or problems with metabolism [37,38]. Clopidogrel must be metabolized by a CYP450 enzyme, typically CYP2C19 and CYP3A4, in order to be active in the body. Individuals with single nucleotide polymorphisms (SNPs) in the genes encoding CYP450 enzymes have decreased metabolization of clopidogrel and. Therefore, decreased activity [11,36,39,40,41]. Additionally, SNPs in genes encoding for P2Y12, COX1, and GPIV have also been found to be associated with non-responsiveness to clopidogrel, as these are involved in platelet activation; it is thought that the identified mutations lead to the disrupted regulation of platelet activation. Clopidogrel is absorbed via a P-glycoprotein transporter in the intestine, and it has been found that those with mutations in this gene are also non-responders to clopidogrel and are at an increased risk of cardiovascular events [35]. While the same regimen for antiplatelet therapy is typically employed to all patients after stent or WEB embolization, the prevalence of non-responders and those with genetic mutations that alter the effect of DAPT details the need for the enhanced monitoring of platelet activity while on antiplatelet therapy.

### 4.5. Aspirin and Ticagrelor

Ticagrelor is in the P2Y12 family and exhibits 40% platelet inhibition within 30 min of administration and peaks at 2 h. Ticagrelor binds reversibly and non-competitively to ADP, unlike clopidogrel and prasugrel which bind irreversibly to receptors. Metabolism is not required for its activation and thus it is not altered by renal function. Ticagrelor is metabolized mainly by CYP3A4 (as are statins) and minimally by CYP3A5. DAPT with ticagrelor is typically used after the failure of clopidogrel, as it has lower resistance rates than clopidogrel; however, its effectiveness has not been well elucidated. Some studies show no difference in complications when using aspirin + clopidogrel vs. aspirin + ticagrelor [42]. Ticagrelor alone has been shown to decrease the risk of death after acute coronary syndrome when compared to clopidogrel alone [43]. Additionally, ticagrelor combined with aspirin is superior to ticagrelor alone in preventing future stroke in those that present with acute coronary syndrome [44]. However, there is an increased risk of bleeding with ticagrelor (13.0%) when compared to clopidogrel (8.3%), and it has the side effect of shortness of breath [45]. In regard to platelet reactivity via TEG mapping, ticagrelor is superior to clopidogrel in regard to platelet inhibition in those with stroke or TIA [46]. Overall, it appears that aspirin + ticagrelor is a safe alternative in those that are non-responders to aspirin + clopidogrel.

### 4.6. Alternative Agents

#### 4.6.1. Prasugrel

An alternative agent that targets the P2Y12 receptor and offers more consistent responses is prasugrel. Its onset is 15–30 min, and it has a duration of action of about five–nine days. In one study, prasugrel was administered to patients who showed high on-treatment platelet reactivity (HTPR) to clopidogrel while undergoing treatment with a pipeline embolization device (PED). Interestingly, no instances of thromboembolic events or bleeding complications were observed in these patients. Likewise, in three different groups of patients who displayed reduced responsiveness to clopidogrel and underwent various neuroendovascular procedures, a switch was made from clopidogrel to either prasugrel or ticagrelor. In one particular study, there was a notable increase in hemorrhagic events among patients receiving prasugrel therapy (*p* = 0.02). However, when a patient with basilar artery perforation was excluded from the data analysis [47], the statistical significance of this finding diminished.

#### 4.6.2. Cilostazol

Cilostazol is another alternative antiplatelet therapy that has an onset of 3–6 h and a duration of action of 48 h. It is a selective inhibitor of phosphodiesterase 3, and the findings of the CSPS2 (Cilostazol Stroke Prevention Study 2) indicate that cilostazol treatment effectively reduces the occurrence of recurrent strokes while causing fewer bleeding events compared to aspirin [48]. The CSPS.com trial (Cilostazol Stroke Prevention Study combination) demonstrated that combination therapy with cilostazol, as opposed to using aspirin or clopidogrel alone, resulted in a reduced recurrence of ischemic stroke in patients during the chronic phase, without an increased risk of bleeding [49,50].

#### 4.6.3. Abciximab, Eptifibatide, and Tirofiban

The receptor responsible for platelet aggregation, primarily facilitated by fibrinogen, is known as glycoprotein IIb/IIIa. Currently, there are three injectable medications that are classified as glycoprotein IIb/IIIa inhibitors: abciximab, eptifibatide, and tirofiban [51,52]. Abciximab is an irreversible inhibitor of the glycoprotein IIb/IIIa receptor with an onset of 2 h and duration of action of 48 h, while eptifibatide and tirofiban are reversible inhibitors of the glycoprotein IIb/IIIa receptors with immediate and 5 min onsets and 2–4 and 3–8 h durations of action, respectively. During neuroendovascular procedures, glycoprotein IIb/IIIa inhibitors are commonly utilized either as an adjunct to DAPT or as a rescue treatment for acute thrombosis. Interestingly, patients who received only intraarterial abciximab did not experience any significant differences in thromboembolic or bleeding complications when compared to those who underwent DAPT in the days leading up to aneurysm coiling. This suggests that the intraarterial infusion of glycoprotein IIb/IIIa inhibitors may be a suitable alternative to DAPT during the procedure. Furthermore, glycoprotein IIb/IIIa inhibitors have proven effective in preventing acute thrombosis in high-risk patients who demonstrate HTPR during neuroendovascular procedures. Many reports in the literature highlight the successful use of intraarterial bolus infusions (usually 12 h after the procedure) of these inhibitors for treating stent or coil thrombosis [11].

**Figure 2:**
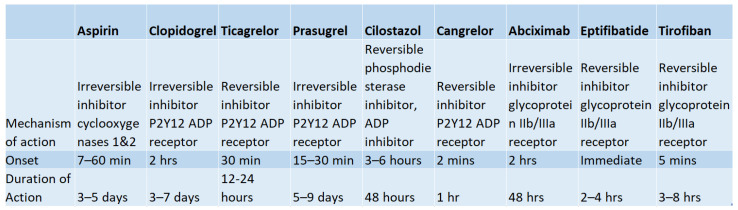
Summary of Properties of Common Antiplatelet Regiments. Mechanisms of action, time of onset, and duration of action of common antiplatelet regimens including aspirin, clopidogrel, ticagrelor, prasugrel, cilostazol, cangrelor, abciximab, eptifibatide, and tirofiban [11].

Figure 2 portrays the mechanisms of action, onset, and durations of action for the antiplatelet therapies discussed above.

## 5. Antiplatelet Therapy Monitoring

### 5.1. PRU Monitoring

The VerifyNow system (Accriva Diagnostics, San Diego, CA, USA) is utilized for the P2Y12 assay, which evaluates the extent of platelet inhibition achieved by P2Y12 inhibitors. The measurement of platelet inhibition is expressed in P2Y12 reaction units (PRU), which are determined by the degree of light transmission through a blood sample [53]. The VerifyNow-P2Y12 assay was created to address the limitations of traditional optical platelet aggregation methods. Its most crucial design element is the utilization of a combination of ADP and prostaglandin E1 within a self-contained assay device to directly assess the impact of clopidogrel on the P2Y12 receptor. ADP is employed to fully activate platelets by binding to both the P2Y1 and P2Y12 platelet receptors, while PGE1 is employed to inhibit the ADP-induced, P2Y1-mediated increase in intracellular calcium levels. This action reduces the contribution from P2Y1 activation and enhances the sensitivity of the assay [54]. The instrument’s validation was conducted in a preclinical environment, where it demonstrated exceptional assay reliability and received FDA approval [55]. A retrospective analysis identified a total of 231 patients who underwent treatment for 248 cerebral aneurysms using a pipeline embolization device (PED). These patients were initiated on DAPT at least 10 days prior to the procedure, and their PRU values were monitored. The analysis, using Youden indices, indicated that maintaining a PRU range from 70 to 150 was deemed optimal, as there were higher chances of complications occurring outside of this range. A PRU value below 60 was found to be a significant predictor of any hemorrhagic complication, while a PRU value exceeding 240 was a significant predictor of any thromboembolic complication, as well as cerebral thromboembolic complications [56,57].

**Figure 3:**
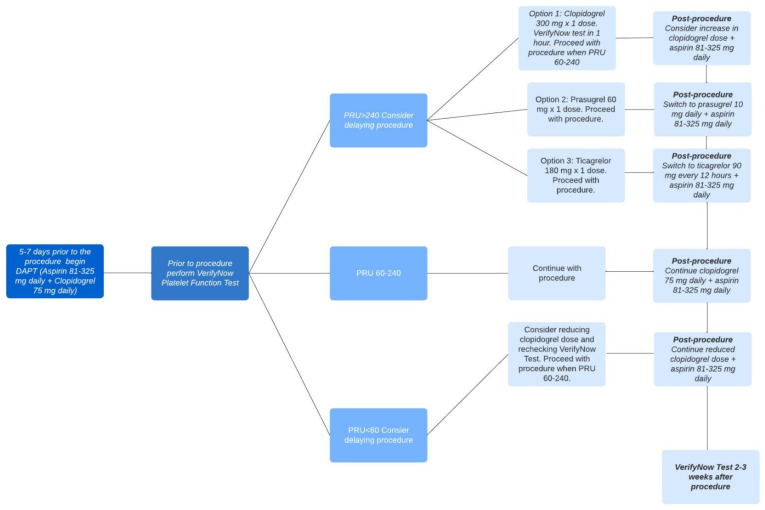
VerifyNow Test Protocol to Adjust Antiplatelet Therapy. There are different options for antiplatelet therapy depending on the PRU measurement prior to the procedure, and these are summarized in this figure. Once the PRU measurement is between 60 and 240, the procedure can be conducted. Following the procedure, there are various post-procedure antiplatelet regimens that can be followed depending on the initial PRU measurement prior to the procedure which are summarized in the figure.

A protocol exists for utilizing the VerifyNow test in patients who are undergoing neuroendovascular procedures. If possible, patients scheduled for neuroendovascular stenting should begin DAPT five–seven days before the procedure. Prior to the procedure, VerifyNow platelet function analysis should be performed for selected patients and procedures. In cases where patients have PRUs exceeding 240 (indicating high on-treatment platelet reactivity or HTPR), there are three potential options available for achieving rapid platelet inhibition. Each option has a corresponding post-procedure treatment plan. These options include administering a single dose of clopidogrel 300 mg along with VerifyNow platelet function analysis within one hour, administering a single dose of prasugrel 60 mg, or administering a single dose of ticagrelor m88 e180 mg. The procedure can proceed when the PRU falls within the range of 60–240. The post-treatment plans include increasing the clopidogrel dose and taking daily aspirin (81–325 mg), taking a daily dose of prasugrel 10 mg along with daily aspirin (81–325 mg), or taking ticagrelor 90 mg every 12 h along with daily aspirin (81–325 mg), respectively. For patients with PRUs below 60 (indicating low on-treatment platelet reactivity or LTPR), clinicians may consider reducing the clopidogrel dose before the procedure. Patients with a PRU within the target range of 60–240 should continue with DAPT as initially prescribed, which consists of taking aspirin (81–325 mg) and clopidogrel (75 mg) daily. This DAPT regimen should be continued after the procedure. VerifyNow test analysis should be performed two–three weeks after the procedure for all of these options [11]. Figure 3 shows an example of a VerifyNow Test protocol to adjust antiplatelet therapy.

### 5.2. Thromboelastography (TEG) Platelet Mapping

**Figure 4:**
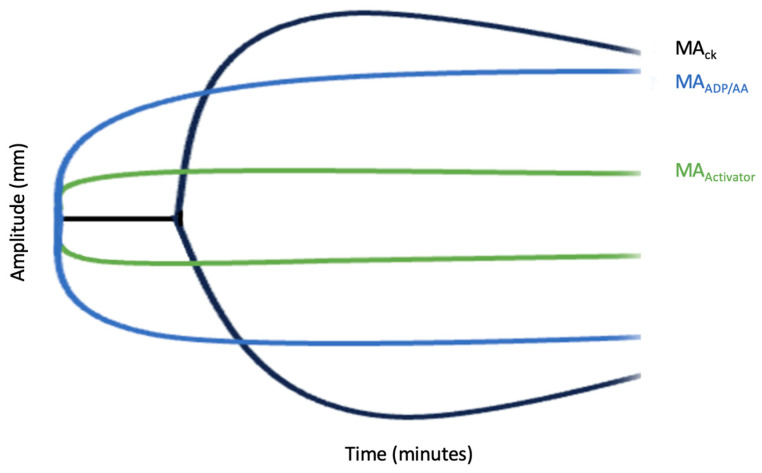
TEG Mapping. This figure shows the activation of platelets based on the conditions amplified at various timepoints. The distance between MA_CK_ and MA_activator_ represents total platelet activation with the distance between MA_CK_ and MA_ADP/AA_ representing platelet inhibition.

TEG platelet mapping is a tool used to assess platelet function in the setting of anticoagulation or antiplatelet therapy [58]. TEG mapping is a faster alternative to routine platelet function testing, typically taking 45 min [58]. The analysis includes evaluating four different platelet activating conditions via determining the maximum amplification (MA)Baseline TEG MA (MA_ck)_, Fibrin TEG MA (MA_activator_), TEG MA_aa_, and TEG MA_ADP_. (Figure 4). The baseline TEG MA (MA_ck)_), also named MA_thrombin_, evaluates the maximal strength of the clot by directly evaluating the fibrin and glycoprotein IIb/IIIa to determine the activated platelet/fibrinogen interaction. Fibrin TEG MA (MA_activator_) also measures the fibrin contribution to clot strength. TEG MA_aa_ measures the platelet activity via thromboxane A2. TEG MA_ADP_ measures the ADP pathway that works in the platelet activation pathway. These factors compose the following equation to determine the percent of inhibition, and typically an inhibition of 70% is considered therapeutic [58]. The values are sometimes altered due to variability in ADP receptors [59].

(1) $$\left[\frac{{\mathrm{MA}}_{\mathrm{AA}}{\;\mathrm{or}\;\mathrm{MA}}_{\mathrm{ADP}}-{\mathrm{MA}}_{\mathrm{Activator}}}{{\mathrm{MA}}_{\mathrm{CK}}-{\mathrm{MA}}_{\mathrm{Activator}}}\times 100\right]=\%\;\mathrm{inhibit}$$

## 6. Bridging Medications

Oftentimes, there is debate on when to initiate antiplatelet therapy post-surgically to avoid post-operative bleeding. IV bridging medications may be used to balance the risk of post-operative bleeding and ischemic events vs. stent thrombosis, with a quicker onset and offset of action than oral medications allowing for titratable doses. This includes cangrelor and tirofiban [60].

### 6.1. Cangrelor

Cangrelor is an ATP analog that inhibits ADP-induced platelet aggregation. It is administered with a 30 µg/kg bolus with a 0.75 µg/kg/min infusion bridging dose. IV cangrelor has an onset of action of 2 min with a half-life of 3–6 min, making it an effective medication for dose adjustment and quick reversal. The platelet activity is restored 1 h after the discontinuation of cangrelor. Additionally, renal and liver function do not affect the pharmacokinetics of cangrelor. Typically, oral antiplatelet therapy is discontinued five days prior to surgery to allow for the regeneration of platelets. Cangrelor can be administered six days prior to surgery and restarted 4–6 h after surgery until an oral antiplatelet regimen can be restarted. Cangrelor can be monitored via PRU and has been shown to have at least 60% platelet inhibition with the bridging dose that normalizes 3 h after discontinuation [58]. In a study regarding neurovascular stent placement for acute ischemic stroke and ruptured and unruptured aneurysms with 37 patients, patients were treated intraprocedurally with a 5 µg/kg bolus and either a 0.75 µg/kg/min or 1 µg/kg/min bridge infusion. PRU monitoring was used and 67% of patients that received the 0.75 µg/kg/min dose were in an acceptable PRU range while 84% of those in the 1 µg/kg/min bridge infusion were in an acceptable range. In this study population of 37 patients, one patient had an intraprocedural stent occlusion and another patient had an occlusion of the artery requiring thrombectomy. No patients experienced hemorrhage. Overall, cangrelor appears to be a safe bridging method for neurovascular stenting due to its quick onset and offset nature and the ability to monitor it with PRU [61].

### 6.2. Tirofiban

Tirofiban, an antiplatelet medication and glycoprotein-IIb/IIIa inhibitor, has been used in the field of neurovascular medicine in two ways: either independently [62] or as a transitional treatment following full-dose intravenous recombinant tissue plasminogen activator (rt-PA) administration [63,64] in patients with acute ischemic stroke (AIS). Some studies have explored the use of tirofiban as an additional therapy during endovascular procedures [65,66] to prevent early re-occlusion and reduce thromboembolic complications [67]. Although there have been reports of an increased risk of secondary intracerebral hemorrhage after ischemic stroke [65,68,69], tirofiban is still utilized in acute stroke cases involving emergency stent implantation, typically as a bridging therapy until dual platelet inhibition with oral clopidogrel and aspirin becomes effective. The rapid onset of action, typically within 20 min, of tirofiban may be beneficial in providing initial platelet inhibition as a bridge before a full antiplatelet effect is achieved with oral antiplatelet medications [70].

One study assessed the effects of a bridging protocol for tirofiban for patients undergoing non-cardiac surgery after DES implantation. They used the following bridging protocol. Clopidogrel was discontinued for five–seven days prior to surgery. Tirofiban was given to patients at a rate of 0.4 μg/kg/min for 30 min, followed by a reduced dose of 0.1 μg/kg/min for patients with normal kidney function. For patients with a creatinine clearance of less than 30 mL/min, the dose of tirofiban was decreased by 50%. The infusion of the glycoprotein IIb/IIIa inhibitor was stopped from four to six hours before surgery. After surgery, a 600 mg initial dose of clopidogrel was administered as soon as it was approved by the surgical team, followed by a once-daily dose of 75 mg. If clopidogrel could not be restarted, it was recommended to resume the glycoprotein IIb/IIIa infusion two hours after surgery and to continue it for up to six hours after the clopidogrel treatment resumed. Throughout and after the surgery, electrocardiograms were conducted for patients with suspected cardiac events, and cardiac biomarkers were measured every 12 h or sooner if necessary based on clinical judgment [71]. Another study used a similar bridging protocol but monitored patients with a 12-lead ECG and blood samples for CK-MB determinations every morning [72].

## 7. Use of AI to Monitor Antiplatelet Therapy

It is crucial to assess the balance between the risks of ischemia and bleeding after stent placement or WEB embolization in neurosurgery and to determine the optimal duration of DAPT accordingly. With varying degrees of adherence to their medication regimen and different levels of platelet inhibition within individuals, it may be possible to use artificial intelligence (AI) to make important decisions regarding when to use antiplatelet therapy. Currently, most research that exists that has studied the use of AI in the prediction of ischemic/bleeding risk exists in the field of cardiovascular medicine, but many of these AI models can potentially be applied to the field of neurosurgery. AI’s predictive capabilities can significantly impact the management of antiplatelet therapy in neurosurgery. The following is a summary of the various ways in which AI can impact the management of antiplatelet therapy in neurosurgery.

**Predicting Ischemic and Bleeding Risks in Neurosurgery:** AI models have been successfully used in cardiovascular medicine to predict ischemic and bleeding risks following procedures like drug-eluting stent (DES) implantation [73,74,75,76,77,78,79]. These models take into account various patient factors, including age, diagnoses, medications, procedures, and DAPT continuation status. AI can adapt and extend these models to neurosurgery settings, helping to predict the risks of ischemic events and bleeding after stent placement or WEB embolization in neuroendovascular procedures. These predictions can guide decisions regarding the timing and duration of antiplatelet therapy.

**Superior Performance of AI Models:** Studies have shown that AI models can outperform traditional clinical tools like the DAPT score in predicting outcomes. For example, AI models demonstrated a higher area under the receiver operating characteristic (AUC) values, a performance metric that is used to evaluate classification models, for predicting ischemia and bleeding compared to the DAPT score [73]. In addition, these models accounted for scenarios that were not addressed by the DAPT score. This improved accuracy can aid neurosurgeons in making more informed decisions about antiplatelet therapy, especially in the 12–30-month window following stent placement.

**Personalized Treatment Plans:** AI models can help create personalized treatment plans based on individual patient profiles and risks. For example, in one study, a machine learning tool known as the PRAISE (Prediction of Adverse Events Following an Acute Coronary Syndrome) predicted death, myocardial infarctions, and major bleeding following an acute coronary syndrome using 25 clinical features that are incorporated during discharge. Based on this information, patients can be categorized into different risk groups (low, intermediate, high), and those at a higher risk can receive closer monitoring and potentially shorter durations of DAPT. This personalized approach enhances patient care and minimizes the risk of complications [80,81].

**AI for Subarachnoid Hemorrhage (SAH), Unruptured Intracranial Aneurysms (UIA), and Chronic Subdural Hematoma Outcomes:** AI has also already been applied to the field of neurosurgery to predict outcomes in conditions like SAH. These predictive models can assess factors such as neurological severity, age, aneurysm location, and size to forecast outcomes [82,83,84,85,86,87,88,89,90,91,92,93,94] with AUC values ranging from 0.70 to 0.90. More recently, deep learning has been employed, which has resulted in an improved prediction accuracy of 0.90 with limited datasets for SAH outcomes [90,93,94]. Two widely recognized radiological scales, namely the Fisher computed tomography (CT) scale [95] and the modified Fisher scale [96], assess the extent of bleeding to forecast delayed cerebral ischemia (DCI) incidence. Additionally, several statistically derived scores [97,98,99] incorporating supplementary factors have been investigated, yielding an average AUC of approximately 0.70 for predicting DCI occurrence [100]. As for AI-based prediction models, they have demonstrated AUC values of around 0.80 [93,101,102,103,104,105] in forecasting DCI occurrence. If we were able to anticipate DCI, we could administer proactive and immediate treatment. Other studies have investigated the potential use of machine learning to predict clinical outcomes in the microsurgical treatment of unruptured intracranial aneurysms (UIA) [106,107,108]. One study offered personalized predictions at the patient level, estimating outcomes such as neurological recovery upon discharge, as well as the likelihood of complications and new neurological issues upon discharge. These predictions are based on readily available preoperative data. The study employed various scoring systems to estimate the risk of aneurysm rupture (PHASES) or growth (ELAPSS), or to directly assess the balance between potential risks and benefits (UIATS) [109]. Methodologies from these studies could be used to anticipate potential complications following UIAs to adjust antiplatelet therapies to respond to them. Additionally, ML models have been used to predict the recurrence risk of chronic subdural hematoma (cSDH) while withholding antiplatelet and anticoagulant agents. This study can also inform the adjustment of antiplatelet regimens according to ischemia/bleeding complication [110].

**Medication Adherence Monitoring:** AI can be used to monitor patient adherence to antiplatelet therapy. For instance, smartphone applications can use AI to confirm medication ingestion through the phone’s camera. Past studies using this technology demonstrated a 67% increase in absolute drug adherence compared to the control [111]. This technology can ensure that patients are adhering to their medication regimen, leading to better outcomes. Similar systems can be adapted for patients undergoing stent or WEB embolization placement, helping to ensure adherence to antiplatelet medications and facilitating communication among healthcare providers.

**Individualized Treatment Plans with PRU and TEG Mapping:** AI platforms can incorporate PRU and TEG mapping to provide individualized treatment plans [111]. This can help to promptly identify non-responders to antiplatelet therapy and adjust their medication regimens accordingly. Through the monitoring of labs, AI has the potential to identify those with suboptimal antiplatelet treatment.

**Patient Engagement and Self-Efficacy:** AI can be used to engage patients in their treatment plans through reminder systems, text messages, and other technologies, as other studies have done in the past [112]. This not only promotes medication adherence but also increases patient self-efficacy in managing their health.

Of course, the commonly discussed challenges associated with integrating AI into clinical practice remain and require attention before widespread clinical adoption [113]. One of these critical challenges revolves around patient privacy concerns, as AI relies on extensive data for algorithm training and sequencing [114]. Striking a balance between privacy protection and data accessibility is essential for sustaining long-term progress in neurosurgical AI [115]. Furthermore, ensuring the quality of data is paramount for meaningful results, emphasizing the importance of effective data implementation in machine learning training [115]. Another issue to consider is the risk of neurosurgeons becoming overly reliant on AI, potentially hindering their skill development, while hardware and software glitches pose the threat of incorrectly directing antiplatelet regimens if not addressed promptly [113,116]. Although artificial intelligence in healthcare has made significant strides, its potential for future advancements remains untapped. The existing innovations have already yielded benefits for patients, but it is crucial for regulatory frameworks to adapt to the rapidly evolving healthcare landscape to proactively address and mitigate potential risks.

In summary, AI’s predictive capabilities can revolutionize the management of antiplatelet therapy in neurosurgery by providing more accurate risk assessments and personalized treatment plans, as well as improved medication adherence. By adapting AI models from cardiovascular medicine and leveraging advanced technologies, neurosurgeons can optimize patient care and outcomes in neuroendovascular procedures (Table 1).

## 8. Conclusions

In conclusion, the field of neurosurgery relies heavily on antiplatelet therapy to manage a range of indications, particularly for stents and WEB embolization. Various antiplatelet regimens are utilized, such as combinations of aspirin and clopidogrel, and aspirin and ticagrelor, as well as alternative agents with diverse mechanisms, onsets, and durations of action, including prasugrel, cilostazol, abciximab, eptifibatide, and tirofiban.

To determine the efficacy of platelet inhibition and adjust dosages based on ischemic or bleeding risks, protocols like PRU monitoring and TEG mapping have been implemented. These methods provide valuable information for optimizing antiplatelet therapy in neurosurgical procedures. Nevertheless, the integration of AI presents an exciting avenue to streamline monitoring processes and offer real-time suggestions for adjusting antiplatelet therapy.

This review summarizes papers which studied the effects of using AI models to predict ischemia/hemorrhage outcomes and hypothesizes that antiplatelet therapy could be adjusted according to these results. The integration of AI into neurosurgery has significant implications for the management of antiplatelet therapy. AI can enhance the risk assessment of adverse outcomes, offer personalized treatment plans based on the categorization of people into various risk groups, improve medication adherence, and increase patient engagement. However, challenges like patient privacy, data quality, and overreliance on AI need to be addressed. Overall, AI has the potential to optimize patient care and outcomes in neuroendovascular procedures, provided that regulatory frameworks adapt to ensure its safe and effective integration. By embracing the integration of AI and antiplatelet therapy, we can strive towards a future where personalized, data-driven treatment approaches become the standard of care in neurosurgery.

## Figures and Tables

**Table 1:** Important articles that address the use of AI to predict clinical outcomes relating to ischemia/hemorrhages.

Author	Year of Publication	Predictive Model Used	Condition Studied	Results
Li et al.; Fan et al. [73,74]	2022, 2019	light gradient boosting machine	Risks of ischemic/bleeding events for patients who had undergone DES implantation	AUC of 0.82 for ischemia and 0.77 for bleeding
Li et al.; Tolles et al. [73,75]	2022, 2016	Logistic regression	Risks of ischemic/bleeding events for patients who had undergone DES implantation	AUC of 0.82 for ischemia and 0.77 for bleeding
Li et al.; Svetnik et al. [73,76]	2022, 2003	Random forest	Risks of ischemic/bleeding events for patients who had undergone DES implantation	AUC of 0.82 for ischemia and 0.77 for bleeding
Li et al.; Cho et al. [73,77]	2022, 2004	Random forest	Risks of ischemic/bleeding events for patients who had undergone DES implantation	AUC of 0.82 for ischemia and 0.77 for bleeding
Li et al.; Choi et al. [73,78]	2022, 2016	RETAIN	Risks of ischemic/bleeding events for patients who had undergone DES implantation	AUC of 0.82 for ischemia and 0.77 for bleeding
D’Ascenzo [81]	2021	PRAISE	ACS syndrome outcomes	AUC for the 1-year risk of MI of 0.74 in the internal validation cohort and 0.81 in the external validation cohort and an AUC for the 1-year risk of major bleeding of 0.70 in the internal validation cohort and 0.86 in external validation cohort
Jaja et al.; Risselada et al.; Abulhasan et al.; Zeiler et al.; Hostettler et al.; Jaja et al.; Witsch et al.; van Donkelaar et al., 2019 [82,83,84,85,86,87,88,89]	2013, 2010, 2018; 2017, 2020, 2018; 2016, 2019	Statistical methods	SAH outcomes	0.70–0.90
Rubbert et al. [91]	2018	Random forests	SAH outcomes	71% accuracy
De Toledo et al. [92]	2009	Random forests	SAH outcomes	0.84 AUC
Katsuki et al.; de Jong et al.; Wang et al. [90,93,94]	2020, 2021, 2022	Deep learning	SAH outcomes	Approximately 0.90 AUC
Ramos et al. [102]	2019	Machine learning	SAH outcomes	AUC 0.74
Meghjani et al. [103]	2021	L2-regularized logistic regression, random forest, and support vector machines models	SAH outcomes	AUC 0.83
De Jong et al. [93]	2021	feedforward artificial neural networks (ffANNs)	SAH outcomes	AUC 0.72
Park et al. [104]	2019	Random kernel	SAH outcomes	AUC 0.77
Savarraj et al. [101]	2021	Machine learning	SAH outcomes	AUC 0.75
Taghavi et al. [105]	2023	Random Forest	SAH outcomes	AUC 0.780
Staartjes et al. [109]	2020	Machine learning models	UIA outcomes	AUC 0.63–0.77
Zanaty et al. [110]	2020	Machine learning models	Recurrence of cSDH	AUC of 0.91

## Data Availability

Not applicable.
